# NKT Cell-TCR Expression Activates Conventional T Cells in Vivo, but Is Largely Dispensable for Mature NKT Cell Biology

**DOI:** 10.1371/journal.pbio.1001589

**Published:** 2013-06-18

**Authors:** J. Christoph Vahl, Klaus Heger, Nathalie Knies, Marco Y. Hein, Louis Boon, Hideo Yagita, Bojan Polic, Marc Schmidt-Supprian

**Affiliations:** 1 Molecular Immunology and Signaltransduction, Max Planck Institute of Biochemistry, Martinsried, Germany; 2 Bioceros, Yalelaan 46, Utrecht, The Netherlands; 3 Juntendo University School of Medicine, Tokyo, Japan; 4 University of Rijeka School of Medicine, Rijeka, Croatia; National Jewish Medical and Research Center/Howard Hughes Medical Institute, United States of America

## Abstract

Natural killer T (NKT) cell development depends on recognition of self-glycolipids via their semi-invariant Vα14i-TCR. However, to what extent TCR-mediated signals determine identity and function of mature NKT cells remains incompletely understood. To address this issue, we developed a mouse strain allowing conditional Vα14i-TCR expression from within the endogenous *Tcrα* locus. We demonstrate that naïve T cells are activated upon replacement of their endogenous TCR repertoire with Vα14i-restricted TCRs, but they do not differentiate into NKT cells. On the other hand, induced TCR ablation on mature NKT cells did not affect their lineage identity, homeostasis, or innate rapid cytokine secretion abilities. We therefore propose that peripheral NKT cells become unresponsive to and thus are independent of their autoreactive TCR.

## Author Summary

Immune system natural killer T (NKT) cells help to protect against certain strains of bacteria and viruses, and suppress the development of autoimmune diseases and cancer. However, NKT cells are also central mediators of allergic responses. The recognition of one's own glycolipid antigens (self-glycolipids) in the thymus via the unique Vα14i T cell receptor, Vα14i-TCR, triggers the NKT cell developmental program, which differs considerably from that of conventional T cells. We generated a mouse model to investigate whether the Vα14i-TCR on mature NKT cells constantly recognizes self-glycolipids and to assess whether this TCR is required for survival and continued NKT cell identity. Switching the peptide-recognizing TCR of a mature conventional T cell to a glycolipid-recognizing Vα14i-TCR led to activation of the T cells, indicating that this TCR is also autoreactive on peripheral T cells or can signal autonomously. But TCR ablation did not affect the half-life, characteristic gene expression or innate functions of mature NKT cells. Therefore, the inherently autoreactive Vα14i-TCR is dispensable for the functions of mature peripheral NKT cells after instructing thymic NKT cell development. Thus the Vα14i-TCR serves a similar function to pattern-recognition receptors, in mediating immune recognition of foreign invasion or diseased cells.

## Introduction

Natural Killer T (NKT) cells represent a subset of T cells in mice and humans that express NK cell markers and recognize a small class of glycolipid (auto-) antigens [1],[2]. Most mouse NKT cells express an invariant Vα14-Jα18 (Vα14i) TCRα rearrangement (Vα24-Jα18 in humans). In principle, all TCRβ-chains are able to pair with this Vα14i-TCR chain [3]. However, the selection of NKT cells by endogenous glycolipids presented by the monomorphic MHC class I-like CD1d induces a strong bias towards TCRs containing Vβ8, Vβ7, or Vβ2 [1],[3], which is abrogated in the absence of selection [3],[4]. Recently, crystallographic analysis demonstrated a conserved binding mode of the NKT cell TCR to various glycolipids, where only germline-encoded residues were in direct antigen contact, reminiscent of innate pattern-recognition receptors [5]. Moreover, several observations suggest that this receptor is inherently auto-reactive [1],[2] and thereby determines NKT cell identity and influences their function. The expression of several inhibitory NK cell receptors on NKT cells was suggested to control their self-reactivity and avoid autoimmune activation [6],[7].

During development in the thymus, the few T cells expressing a Vα14i-TCR are selected upon recognition of self-lipids on double-positive thymocytes. Although several good candidates have been put forward [8]–[10], the exact nature of the selecting glycolipids remains controversial. Homotypic interactions involving the SLAM family (SLAMf) receptors 1 and 6 are additionally required for NKT cell differentiation [11]. Auto-reactive activation during thymic selection is thought to induce a substantially stronger TCR stimulus in comparison to that during the development of conventional T cells [12],[13]. As a consequence, expression of the transcription factors Egr1 and Egr2 is strongly increased [13], which in turn directly induce PLZF, the key transcription factor controlling NKT cell differentiation, migration, and functions [13].

Interestingly, the homeostatic proliferation of NKT cells after adoptive transfer was similar in CD1d-deficient and wild-type mice, indicating that this process is mostly cytokine-driven and does not depend on continued TCR-mediated self-lipid-recognition [14],[15]. However, as the transferred cells contained CD1d, a role for antigen could not be completely excluded. In addition, tonic antigen-independent TCR signals might contribute to NKT cell maintenance and phenotype. During immune responses, NKT cell activation depends mostly on two parameters: engagement of the TCR and the presence of proinflammatory cytokines released from antigen-presenting cells activated by innate immune pathways such as toll-like receptor (TLR) signals. Lipids derived from different bacteria [16]–[19] were shown to directly activate mouse and human NKT cells in a TLR- and IL-12-independent manner, and NKT cells are required for productive immune responses against these pathogens. NKT cells can also be activated indirectly through cytokines such as IL-12, IL-18, or type I interferons (IFNs) [20]. However, it remains controversial whether, depending on the strength of the cytokine signal, weak responses to self-antigens presented by CD1d are an additional obligate requirement. In one study, CD1d-dependent signals were found to be necessary for full NKT cell activation in response to all tested pathogens [20]. In contrast, others reported that IL-12-dependent NKT cell activation after LPS injection [21] or MCMV infection [22] is independent of either foreign or self-glycolipid antigen presentation by CD1d.

Upon activation, the most distinguishing feature of NKT cells is their ability to rapidly produce and secrete large amounts of cytokines (Th1 and Th2 cytokines, among others). Their fast, effector-like response could be based on steady-state expression of cytokine mRNA in mice [23],[24] that was suggested to be a consequence of tonic self-reactive activation [2]. Recently, it was reported that human NKT cells do not constitutively express cytokine mRNAs. Instead, rapid cytokine-induced innate IFNγ production by NKT cells was suggested to rely on obligate continuous recognition of self-lipids, which retains histone acetylation patterns at the *IFNG* locus that favor transcription [25]. Another characteristic feature of NKT cells, their surface marker expression reminiscent of memory or recently activated T cells, was also connected to their inherent autoreactivity [2].

To thoroughly address the open questions regarding the nature and importance of TCR signaling for NKT cells, we generated a novel mouse model that allowed us to study the extent of Vα14i-TCR-mediated auto-antigen recognition in the periphery and its relevance for NKT cell identity. Furthermore, we monitored the fate of NKT cells after TCR ablation. Our results prove the inherent self-reactivity of the NKT cell TCR and demonstrate that although essential for positive selection, tonic TCR signaling is not required for NKT cell homeostasis, lineage identity, and rapid cytokine secretion.

## Results

### Correct Timing and Endogenous Control of Vα14i-TCR Expression Produces Large Numbers of Bona Fide NKT Cells

**Figure 1:**
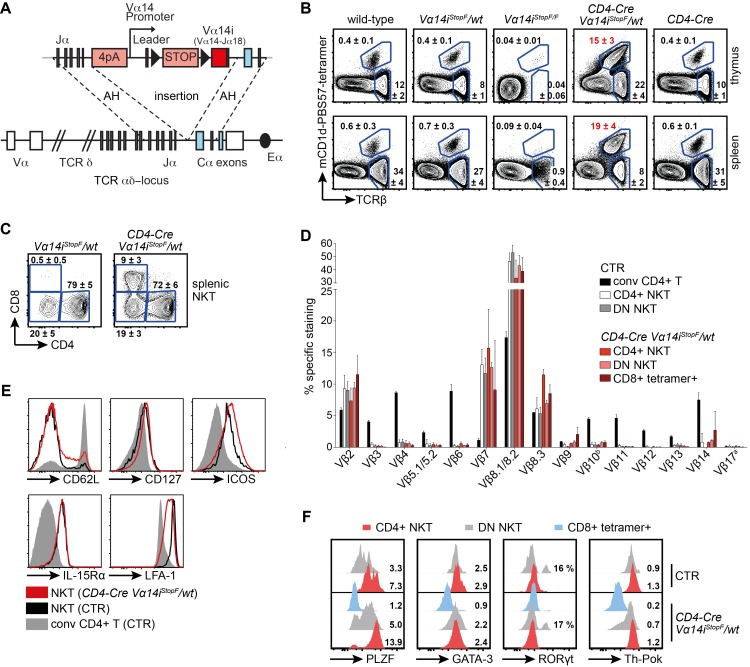
The Vα14i-TCR knock-in mouse produces large numbers of correctly selected, bona fide NKT cells. (A) Schematic representation of the knock-in transgene. The *Vα14* promoter, *loxP* (triangle)-flanked STOP cassette, and pre-rearranged *Vα14i* (Vα14-Jα18, red square) sequences were inserted 3′ of *Jα1* and 5′ of the first *Cα* exon (coding exons are highlighted in blue); 4pA = 4 SV40 polyadenylation sites. AH, arms of homology. *Eα*, enhancer (black oval). (B) Representative proportions of NKT cells and conventional T cells of total lymphocytes in thymus and spleen. Numbers indicate mean percentages ± SD of at least seven age-matched mice per genotype. (C) Representative proportions of splenic CD4+, CD8+, and DN (CD4− CD8−) NKT cells. Numbers indicate mean percentages ± SD of seven mice per genotype. (D) The Vβ repertoires of splenic NKT cells of the indicated genotypes. Bars indicate means and error bars SD of three independent experiments. (E) Representative flow cytometric analysis of the indicated cell-surface proteins on conventional CD4+ T cells and NKT cells. (F) Intracellular flow cytometric staining of PLZF, GATA-3, ROR-γt, and Th-POK in the depicted NKT cells. Numbers indicate means of the median fluorescence intensities (MFIs), normalized to CD4+ tetramer− T cells of CTR animals, or percentage of ROR-γt+ cells among DN NKT cells; calculated from three animals per genotype. Histograms are representative of three independent experiments with eight mice in total. Throughout the figure, NKT cells were gated as tetramer+ TCRβ+, conventional (conv) T cells as tetramer− TCRβ+; CTR, *CD4-Cre* or *Vα14i^StopF^/wt*.

**Figure 2:**
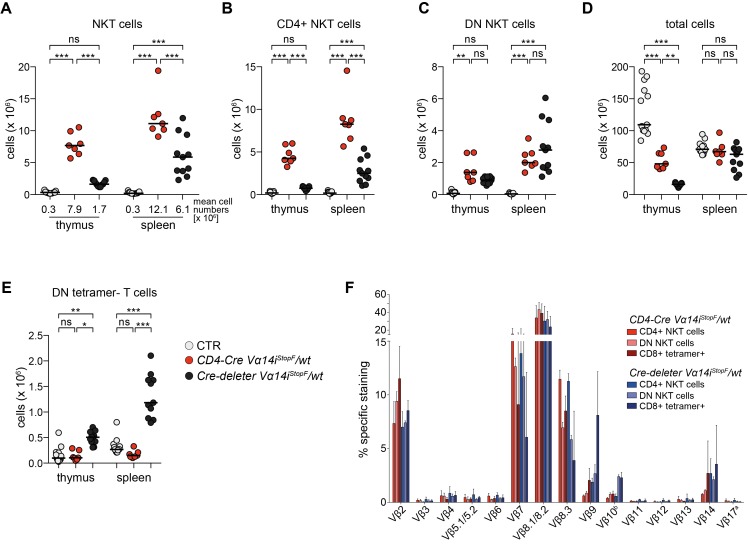
Premature Vα14i-TCR expression impairs NKT and conventional T cell development. (A–E) Absolute cell numbers in thymus and spleen of 7–13 mice of the indicated genotypes: NKT cells (A), CD4+ NKT cells (B), DN NKT cells (C), total cells (D), and DN tetramer− T cells (E). Bars indicate medians. *** *p*<0.001; ** *p*<0.01; * *p*<0.05; ns, not significant; one-way ANOVA. (A) Mean cell numbers are depicted below the scatter blot. (F) The Vβ repertoires of splenic NKT cells of the depicted animals. Data for *CD4-Cre Vα14i^StopF^/wt* are the same as shown in Figure 1D. Bars indicate means and error bars SD of 3–4 mice per genotype of 3–4 independent experiments. Throughout the figure, NKT cells were gated as tetramer+ TCRβ+, conventional (conv) T cells as tetramer− TCRβ+; CTR, *CD4-Cre* or *Vα14i^StopF^/wt*.

In order to produce large numbers of NKT cells in a physiological manner and to manipulate the expression of the semi-invariant Vα14i-TCR in a conditional fashion, we generated *Vα14i^StopF^* knock-in mice. To this end we cloned a productive *Vα14-Jα18* rearrangement, including the *Vα14* leader exon, intron and 1.8 kb of upstream regulatory sequence, and 0.2 kb intronic sequence downstream of *Jα18*. These elements were inserted by homologous recombination 3′ of *Jα1* upstream of the *Cα* constant region of the *Tcrα* locus (Figure 1A). Expression of putative upstream rearrangements is aborted by four SV40 polyA sites at the 5′ end of the construct, and expression of Vα14i is rendered conditional through a loxP-flanked STOP cassette. We obtained over 80% (271 of 325) homologous recombinant ES cell clones during gene targeting, indicating an unusually high targeting efficiency of our construct (Figure S1A). The development of conventional T and NKT cells, identified by staining with mouse CD1d-PBS57-tetramers (tetramer+), occurs unperturbed in *Vα14i^StopF^/wt* heterozygous mice. In homozygous *Vα14i^StopF/F^* mice, T cell development is abolished due to transcriptional termination of TCRα expression before the Cα exons (Figure 1B). We bred *Vα14i^StopF^* to *CD4-Cre* mice, in order to express the inserted Vα14i-chain in double-positive thymocytes, mimicking the physiological timing of TCRα-chain rearrangement and expression [26],[27]. On average 23 times more thymic and 43 times more splenic NKT cells were generated in these, compared to wild-type mice (Figures 1B and 2A–E). Around 9% of the tetramer+ T cells in *CD4-Cre Vα14i^StopF^/wt* mice expressed the CD8 co-receptor (over 80% as CD8αβ heterodimer; Figures 1C and S1B,C), which is also expressed by some human NKT cells, but normally not in mice [28]. The proportions of CD4− CD8− double negative (DN) and CD4+ cells were comparable between transgenic (tg) and wild-type NKT cells (Figure 1C). Furthermore, the tgNKT cells were largely comparable to wild-type NKT cells with respect to Vβ-chain bias (Figure 1D) and surface phenotype (Figure 1E). Finally, we found that NKT cells from *CD4-Cre Vα14i^StopF^/wt* animals expressed the critical transcription factors promyelocytic leukemia zinc finger (PLZF), GATA binding protein 3 (GATA-3), and T-helper-inducing POZ/Krüppel-like factor (Th-POK) (Figure 1F) [28],[29]. Interestingly, we also detected a substantial proportion of the recently described NKT17 subset in the transgenic animals. These DN NK1.1^−^ NKT cells express the transcription factor ROR-γt and were shown to produce the cytokine IL-17 upon activation (Figure 1F) [29],[30].

### Timing of Transgenic Vα14i-TCR Expression Is Critical for Normal NKT Cell Development

Premature TCRα expression leads to aberrant T cell development in transgenic mouse models [26],[27]. To directly compare the consequence of premature to CD4-Cre-mediated timely Vα14i-TCRα-chain expression in our knock-in approach, we bred our mice to a germline *Cre-deleter* strain (*Nestin-Cre*) [31]. Compared to CD4-Cre-induced Vα14i-TCRα-chain expression, premature expression in *Cre-deleter Vα14i^StopF^/wt* led to significantly reduced numbers of NKT cells in thymus and spleen, especially of CD4+ NKT cells (Figure 2A–C). In addition, we found reduced thymocyte counts and a significant increase of most likely lineage-“confused” DN (CD4− CD8−) tetramer-negative T cells (Figure 2D,E). In fact *Cre-deleter Vα14i^StopF^/wt* mice strongly resemble the “first generation” Vα11 promoter-driven (Vα11p) *Vα14i* transgenic mice in these respects (Table S1) [32]. Moreover, in *Cre-deleter Vα14i^StopF^/wt* mice, we observed increased proportions of Vβ9-, Vβ10-, and Vβ14-containing Vα14i-TCRs, which can recognize α-GalCer-loaded tetramers, but most likely not endogenous self-glycolipids [3],[4], pointing to perturbed positive selection (Figure 2F). *CD4-Cre Vα14iStop^F^/wt* mice produce more NKT cells than any of the previously reported models, including mice with a *Vα14i* allele derived from a NKT cell nuclear transplantation experiment [11],[32]–[35]. A comparison of different *Vα14i*-transgenic models demonstrates that both the correct timing and endogenous control of TCR expression control favor NKT cell development (Table S1). Our analyses therefore showed that physiological timing of Vα14i-TCRα-expression at endogenous levels in *CD4-Cre Vα14i^StopF^/wt* mice contributes to the production of large numbers of correctly selected, bona fide NKT cells.

### NKT Cell Maturation in *CD4-Cre Vα14i^StopF^/wt* Animals

**Figure 3:**
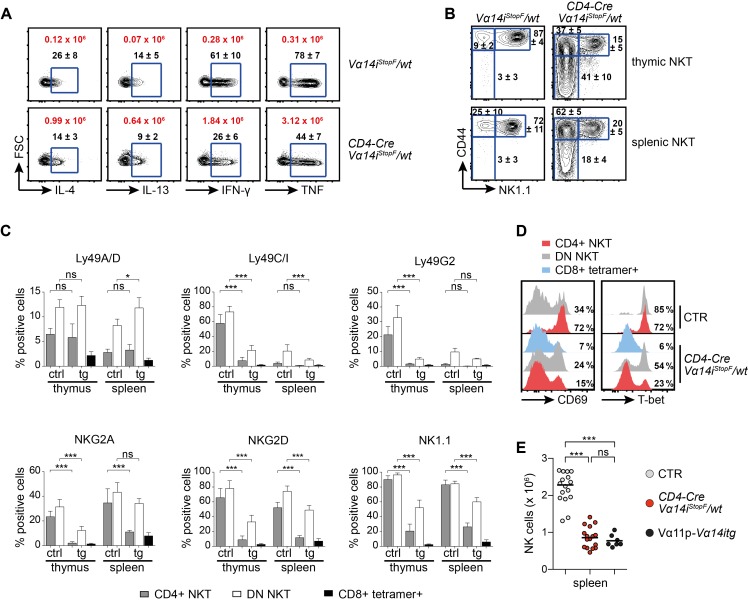
NKT cell overproduction affects their maturation and NK cell homeostasis. (A) Intracellular IL-4, IL-13, IFN-γ, and TNF expression of splenic CD4+ NKT cells isolated from the depicted animals 90 min after αGalCer injection. Cells were stained directly ex vivo without addition of brefeldin or monensin. Black numbers indicate mean percentages ± SD, and red numbers indicate mean total NKT cell counts expressing the respective cytokine. Data are from three animals per genotype; FSC, forward scatter. (B) Representative proportions of stage 1 (CD44^low^ NK1.1^low^), stage 2 (CD44^high^ NK1.1^low^), and stage 3 (CD44^high^ NK1.1^high^) thymic and splenic NKT cells. Numbers indicate mean percentages ± SD of 10 mice per genotype. (C) Flow cytometric analysis of the depicted markers on thymic and splenic, transgenic, and control NKT cells. Bars indicate means and error bars SD calculated from 4–7 mice. (D) Extracellular and intracellular flow cytometric stainings of CD69 and T-bet in the depicted NKT cell subpopulations. Numbers in representative histogram indicate percentage of CD69^high^ or T-bet+ cells among the indicated NKT cells calculated from eight animals per genotype (CD69) or three animals per genotype (T-bet). Histograms are representative of at least three independent experiments with each at least seven mice in total. (E) Absolute splenic NK cell numbers (NK1.1+ TCRβ− tetramer–) of age-matched 6–12-wk-old animals (7–16 per genotype). Bars indicate medians. *** *p*<0.001; ns, not significant; one-way ANOVA. Throughout the figure, NKT cells were gated as tetramer+ TCRβ+, conventional (conv) T cells as tetramer− TCRβ+; CTR, *CD4-Cre* or *Vα14i^StopF^/wt*.

To test the functionality of our transgenic NKT cells, we injected *CD4-Cre Vα14i^StopF^/wt* mice with the NKT cell ligand α-Galactosylceramide (α-GalCer) and determined their cytokine production directly ex vivo. The transgenic NKT cells were able to mount a rapid and robust cytokine response. Although a reduced proportion of transgenic NKT cells responded, in absolute cell numbers there was a 6–10-fold increase compared to wild-type NKT cells (Figure 3A). We did not observe significant steady-state cytokine production by transgenic or control NKT cells, and we detected only minor increases in cytokine levels in the serum of some of these mice (Figure S1D). Since cytokine production also varies with NKT cell maturation, we analyzed NKT cell development in *CD4-Cre Vα14i^StopF^/wt* mice in more detail. This revealed a strong bias toward immature fractions in the thymus, due to the dramatic increase in NKT cell progenitors. In the periphery, 20% of NKT cells fully matured, as judged by the expression of NK1.1 and other NK cell markers (Figure 3B,C). This view is further supported by the reduced proportion of CD69 and T-bet-expressing NKT cells in *CD4-Cre Vα14i^StopF^/wt* compared to wild-type mice (Figure 3D). The expression of both CD69 and T-bet strongly correlated with NK1.1 surface levels (Figure S1E,F). This also explains the higher intracellular PLZF expression in CD4+ and DN NKT cells of *CD4-Cre Vα14i^StopF^/wt* animals in comparison to control animals (Figure 1F), as it was shown that PLZF expression is downregulated during NKT cell development [36]. Reduced maturation seems to be a common feature in mice with overabundance of NKT cells (Figure S1G and Table S1) [33]. Indeed, a comparison of different Vα14i-tg mice suggests that independently of the total number of NKT cells generated, the size of the homeostatic niche for mature NKT cells appears to be around two million cells (Table S1).

IL-15 is critical for the final maturation of NKT cells [37] and together with IL-7 required for their peripheral maintenance [14],[38]. NKT cells compete with NK cells for these resources [38]. The halved number of NK cells in *CD4-Cre Vα14i^StopF^/wt* mice (Figure 3E) suggests that the availability of these and maybe other cytokines might be insufficient due to the dramatically increased NKT cell numbers. The fact that a similar effect was observed in Vα11p-*Vα14itg* mice (Figure 3E) underscores this notion. These results let us conclude that while large amounts of NKT cells can be produced in mice, depending on the mode of Vα14i expression, the number of fully mature NKT cells is restricted by homeostatic constraints, some of which are shared with NK cells.

### The Exchange of the Endogenous TCR Repertoire for a Vα14i-Restricted One on Mature Conventional T Cells Leads to a Significant Population of Tetramer+ T Cells

The strong self-lipid-induced TCR stimulus that early NKT cell progenitors receive in the thymus can be visualized through high GFP expression under the control of the *Nur77* gene locus, reporting TCR signal strength [12]. However, the subsequent loss of GFP in mature NKT cells suggests that these cells are either not exposed to or not responsive to self-antigens. In order to answer this question and to study NKT cell TCR-autoreactivity in the periphery, we investigated the consequences of Vα14i-TCR signals for conventional naïve T cells. We wondered whether Vα14i-TCR expression on naïve T cells, lacking inhibitory receptors and generally a NKT cell “identity”, would lead to activation upon (self-)lipid recognition and what cellular fate(s) are elicited by such activation.

**Figure 4:**
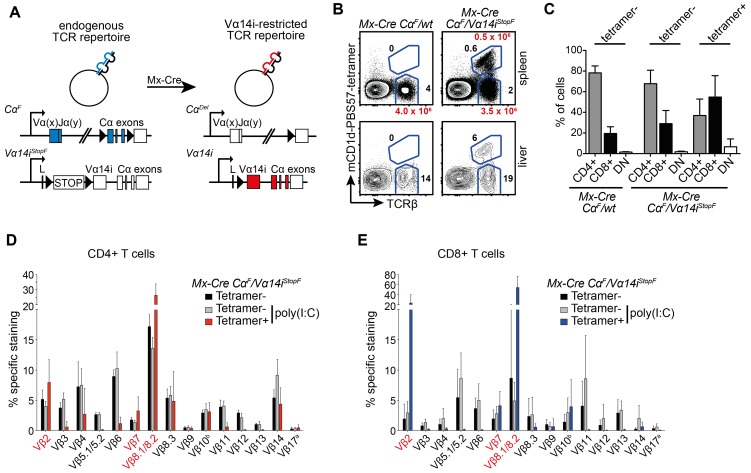
TCR switch on mature conventional T cells. (A) Genetic set-up of the TCR switch experiment. In *Mx-Cre Cα^F^/Vα14i^StopF^* mice, the endogenous TCRα-chains (Vα(x)Jα(y)) are exclusively expressed from the *Cα^F^* allele. Cre-mediated recombination leads to termination of expression from the *Cα^F^* allele, and simultaneous start of expression of the Vα14i-TCRα-chain from the *Vα14i^StopF^* allele. (B) T-cell-deficient mice were reconstituted with NKT cell-depleted splenocytes of the indicated genotypes. After 2 wk, the TCR switch was induced by poly(I:C) injection. Eight weeks later, percentages of tetramer+ and tetramer− T cells (TCRβ+) were analyzed in spleen and liver. Black numbers indicate percentages of total lymphocytes, red numbers absolute cell number calculated from 9–17 animals. (C) Bars indicate means and SD (error bars) of CD4+, CD8+, or DN (CD4− CD8−) cells among tetramer− and tetramer+ T cells, calculated from at least nine mice per genotype. (D, E) The Vβ repertoires of the depicted splenic CD4+ (D) or CD8+ (E) T cell subsets isolated from T-cell-deficient animals that received NKT cell-depleted *Mx-Cre Cα^F^/Vα14i^StopF^* splenocytes. Some of these mice were injected with poly(I:C) 2 wk later to induce the TCR switch. Eight weeks after poly(I:C) injection, the Vβ repertoires were analyzed. Data represent means and SD (error bars) of two independent experiments with a total of three mice (tetramer− without poly(I:C) injection) or eight mice (poly(I:C) injected) per T cell population. Vβs typical for glycolipid selection of NKT cells are highlighted in red.

To this end, we generated mice enabling us to exchange the endogenous TCR-repertoire present on naïve peripheral T cells for a Vα14i-restricted TCR repertoire. The induction of Cre expression in *Mx-Cre Cα^F^*/*Vα14i^StopF^* mice inactivates the *Cα^F^* allele and simultaneously turns on the *Vα14i^StopF^* allele, leading to substitution of endogenous TCRα-chains with the Vα14i TCRα-chain (Figure 4A). As mentioned above, the Vα14i-chain can pair with all TCRβ-chains [3], although only Vβ2-, Vβ7-, and Vβ8-containing Vα14i-TCRs can recognize endogenous lipids such as iGb3 [3],[4]. Since TCRs containing one of these Vβ-chains constitute approximately 30% of the CD4+ and CD8+ peripheral T cell pool (Figure 1D and unpublished data), we predicted that our genetic switch experiment should generate sufficient numbers of T cells able to recognize self-lipids.

In *Mx-Cre* transgenic mice, Cre expression can be induced through injection of dsRNA, such as poly(I:C) [39]. However, low-level “leaky” recombination occurs also in absence of an inducer [39],[40], leading to increased numbers of tetramer+ T cells in naive *Mx-Cre Cα^F^/Vα14i^StopF^* mice (Figure S2A). Therefore, splenocytes were depleted of tetramer+ T cells by magnetic cell separation (MACS, Figure S2A), and 20×10^6^ purified cells were injected intravenously (i.v.) into recipient animals lacking conventional αβ T cells and NKT cells (*Cα^−/−^* or *Vα14i^StopF/F^*). After cells were allowed to engraft for 2 wk, the TCR switch was induced by poly(I:C) injection. Importantly, except for a short-term activation of the immune system, poly(I:C) injection in *Mx-Cre* mice per se has no significant long-lasting effect on peripheral conventional T cells [40],[41] or on the number and phenotype of NKT cells (unpublished data). To definitely exclude any effect of poly(I:C) injection on our results, we waited 2–4 mo before analyzing the animals after the induced TCR switch.

We found significant numbers of tetramer+ CD4+ and CD8+ T cells as a result of this switch experiment (Figure 4B–E). “Unloaded” tetramers did not stain these cells, demonstrating that they were not reactive against CD1d itself (Figure S2B). The TCR-switched tetramer+ T cells were predominantly enriched in cells expressing Vβ-chains that are associated with high avidity auto-antigen binding: Vβ2, Vβ8.1/8.2, and Vβ7 (Figure 4D,E) [3],[4],[42]. The exceptions were CD8+ TCR-switched tetramer+ T cells, in which Vβ7-expressing cells were not enriched. The bias toward tetramer+ CD8+ T cells (Figure 4C) is most likely due to more efficient Mx-Cre-mediated recombination in these cells [40].

### Sterile Inflammation in Mice Containing TCR-Switched T Cells

**Figure 5:**
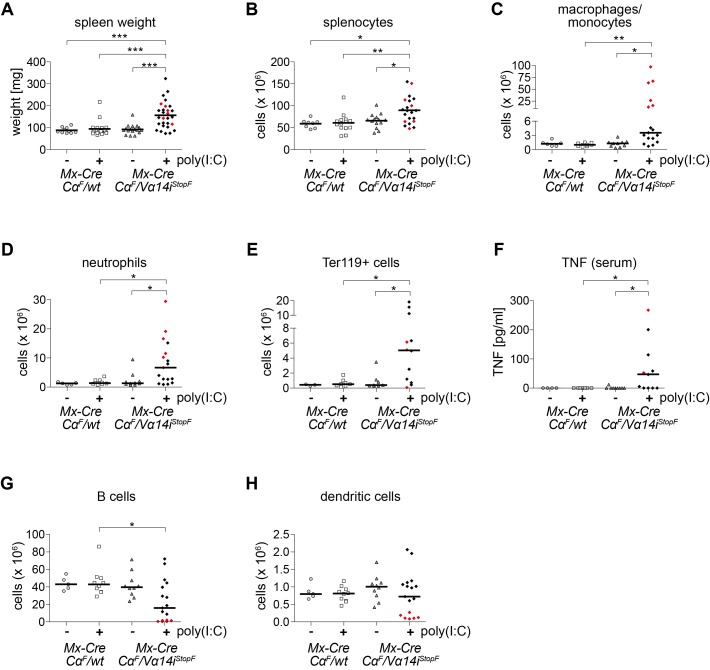
Signs of sterile inflammation in mice harboring TCR-switched T cells. T-cell-deficient mice were reconstituted with NKT-cell-depleted splenocytes of the indicated genotypes. Spleen weight (A), absolute splenic cell numbers (B–E, G, H), and serum TNF levels (F) of 3–28 mice per genotype were determined 8 wk after poly(I:C) administration where indicated. Bars indicate medians. Red points show six animals with near absence of B cells and dendritic cells. (B) Total splenocytes; (C) Macrophages/monocytes (Mac1+ Gr1^int^ SiglecF−); (D) Neutrophils (Mac1+ Gr1^high^ SiglecF−); (E) Erythroblasts (Ter119+); (G) B cells (B220+ TCRβ−); (H) Dendritic cells (CD11c+). *** *p*<0.001; ** *p*<0.01; * *p*<0.05, one-way ANOVA.

Animals containing TCR-switched tetramer+ T cells, but not controls, displayed splenomegaly (Figure 5A,B), characterized by increased numbers of macrophages/monocytes, neutrophils, and Ter119+ erythroid progenitor cells, suggesting an inflammatory state (Figure 5C–E). In line with these findings, we could detect elevated serum TNF in more than half of these mice (Figure 5F). Elevated levels of other cytokines, such as IL-2, IL-4, IL-5, IL-6, IL-10, IL-17, and IFN-γ, were not found in the sera of these mice (unpublished data). Interestingly, we found that 6 (highlighted in red throughout the figure) of 17 spleens containing TCR-switched T cells were almost completely devoid of B cells (Figure 5G) as well as dendritic cells (DCs, Figure 5H), which present lipid antigens to NKT cells via CD1d [1]. Furthermore, tetramer- “conventional” T cells were also strongly reduced in these animals (unpublished data). Together, these results suggest that induced expression of the Vα14i-TCR on conventional naïve T cells causes sterile inflammation, possibly due to autoimmune activation.

### Vα14i-TCR Signaling Induces Cellular Activation, But Not NKT Cell Differentiation of TCR-Switched Tetramer+ T Cells

**Figure 6:**
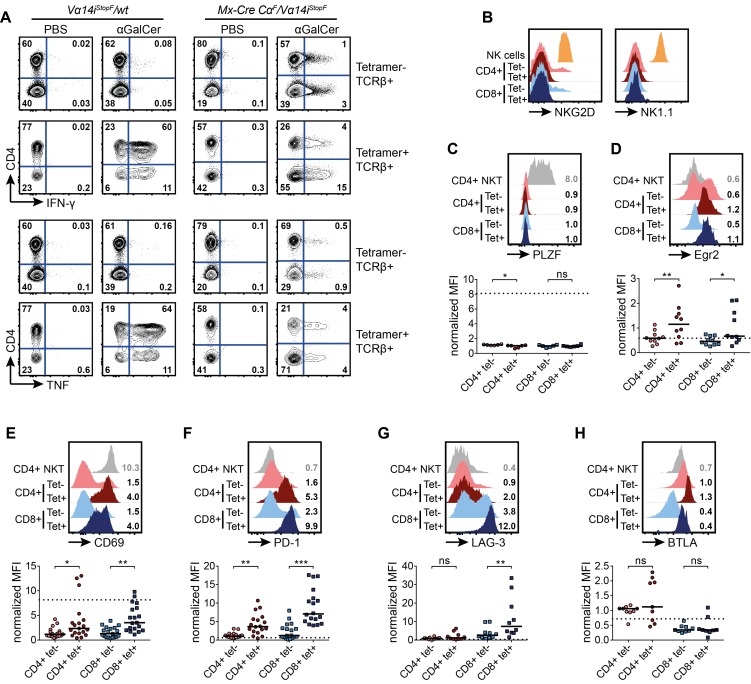
TCR-switched tetramer+ T cells display an activated/exhausted phenotype, but no signs of NKT cell differentiation. T-cell-deficient mice were reconstituted with NKT-cell-depleted splenocytes of the indicated genotypes. The TCR switch was induced by poly(I:C) administration. Eight weeks later, the animals were analyzed. (A) Expression of intracellular IFN-γ or TNF ex vivo 90 min after αGalCer injection of the indicated mice. Data are representative of two independent experiments with two animals each. (B) Representative histograms of flow cytometric analyses. Surface expression of NKG2D and NK1.1 on T cells (TCRβ+) of the indicated surface phenotypes in comparison to NK cells (NKG2D+ TCRβ− CD5− or NK1.1+ TCRβ− CD5−) are shown. Histograms are representative for at least three independent experiments with at least one mouse each. (C–H) Representative histograms of flow cytometric analyses. T cells (TCRβ+) of the indicated surface phenotypes, and of wild-type splenic CD4+ NKT cells, are shown. Numbers in representative histograms indicate means of the median fluorescence intensities (MFIs), normalized to CD4+ tetramer− T cells of animals that received NKT-cell-depleted *Mx-Cre Cα^F^/wt* splenocytes, 8 wk after poly(I:C) injection. Means were calculated from 6–25 mice. Scatter plots display normalized MFI. Bars indicate medians. Dotted lines indicate medians of the median fluorescence intensities of control CD4+ wild-type NKT cells calculated from 2–6 mice. (C, D) Intracellular PLZF (C) and Egr2 (D) expression. (E–H) Extracellular expression of CD69 (E), PD-1 (F), LAG-3 (G), BTLA (H); *** *p*<0.001; ** *p*<0.01; * *p*<0.05; ns, not significant; one-way ANOVA.

The appearance of tetramer+ cells displaying a Vβ bias similar to antigen-selected NKT cells, together with signs of inflammation upon TCR switch and the absence of CD1d-expressing B cell and DCs in some cases, suggested auto-antigen-mediated activation of TCR-switched cells. To verify that the newly assembled Vα14i-TCR on conventional T cells is functional, we injected recipients of *Mx-Cre Cα^F^/Vα14i^StopF^* and control cells with α-GalCer or PBS 2 mo after switch induction. Ninety minutes after α-GalCer, but not PBS, injection, CD4+ and CD8+ tetramer+ T cells produced IFN-γ and TNF (Figure 6A), demonstrating the functionality of the newly assembled Vα14i-TCR. In comparison to NKT cells from wild-type or *CD4-Cre Vα14i^StopF^/wt* animals, a smaller proportion of tetramer+ T cells produced cytokines (Figures 6A and S2C). Tetramer+ TCR-switched T cells could also be activated in vitro through α-GalCer-pulsed A20 cells overexpressing CD1d (unpublished data) [43].

To study the consequences of Vα14i-TCR expression on tetramer+ TCR-switched T cells in more detail, we analyzed their surface phenotype and transcription factor expression. Absence of NK cell markers (Figures 6B and S2D) and PLZF expression (Figure 6C) indicated that the Vα14i-TCR signals are not sufficient to induce NKT cell differentiation of mature conventional T cells. However, the TCR-switched tetramer+ T cells expressed significantly higher levels of Egr2 in comparison to tetramer− T cells in the same animals (Figure 6D), suggesting that the switched cells receive stronger TCR signals [13]. TCR-switched T cells showed further signs of cellular activation, as they expressed elevated levels of CD69 (Figure 6E). Interestingly, these T cells displayed also significantly increased surface levels of PD-1, LAG-3, and less frequently, BTLA and TIM-3, which is typical of exhausted/anergic cells (Figure 6F–H and unpublished data) [44],[45].

To test whether exhaustion/anergy of tetramer+ TCR-switched T cells prevented a more dramatic form of autoimmune inflammation, we injected mice with PD-L1 and PD-L2 blocking or control antibodies twice a week for 4 consecutive weeks, starting 2 d before switch induction. The administration of these blocking antibodies has previously been shown to efficiently prevent anergy induction of conventional T as well as NKT cells, and to partially reverse the exhaustion of CD8+ T cells [44],[45]. However, we did not observe any dramatic differences in spleen weight or cellularity, or signs of increased inflammation, between animals receiving PD-L blocking or control antibodies (unpublished data). In response to PD-1 blockade, other inhibitory receptors such as LAG-3, BTLA, or TIM-3 might control the TCR-switched T cells.

Taken together, our results showed that expression of the Vα14i-TCR on mature conventional T cells is not sufficient to induce a NKT cell differentiation program. Still, it is likely that Vα14i-TCR signals induce auto-antigen-mediated activation, possibly to the point of exhaustion. We therefore present strong evidence that the Vα14i-TCR can constitutively recognize self-lipids in the naïve steady state situation in vivo.

### Maintenance of Mature NKT Cells Is TCR-Independent

The evidence for autoreactivity of the Vα14i-TCR on mature peripheral T cells raised the old but still not completely resolved question whether and to what extent interactions with self-lipid-presenting APCs are required for NKT cell maintenance, cellular identity, and function. In order to evaluate the importance of constitutive TCR expression and signaling for NKT cells directly in vivo and for long periods of time, we ablated the TCR on mature T cells using poly(I:C) injection of *Mx-Cre Cα^F/F^* mice [40].

**Figure 7:**
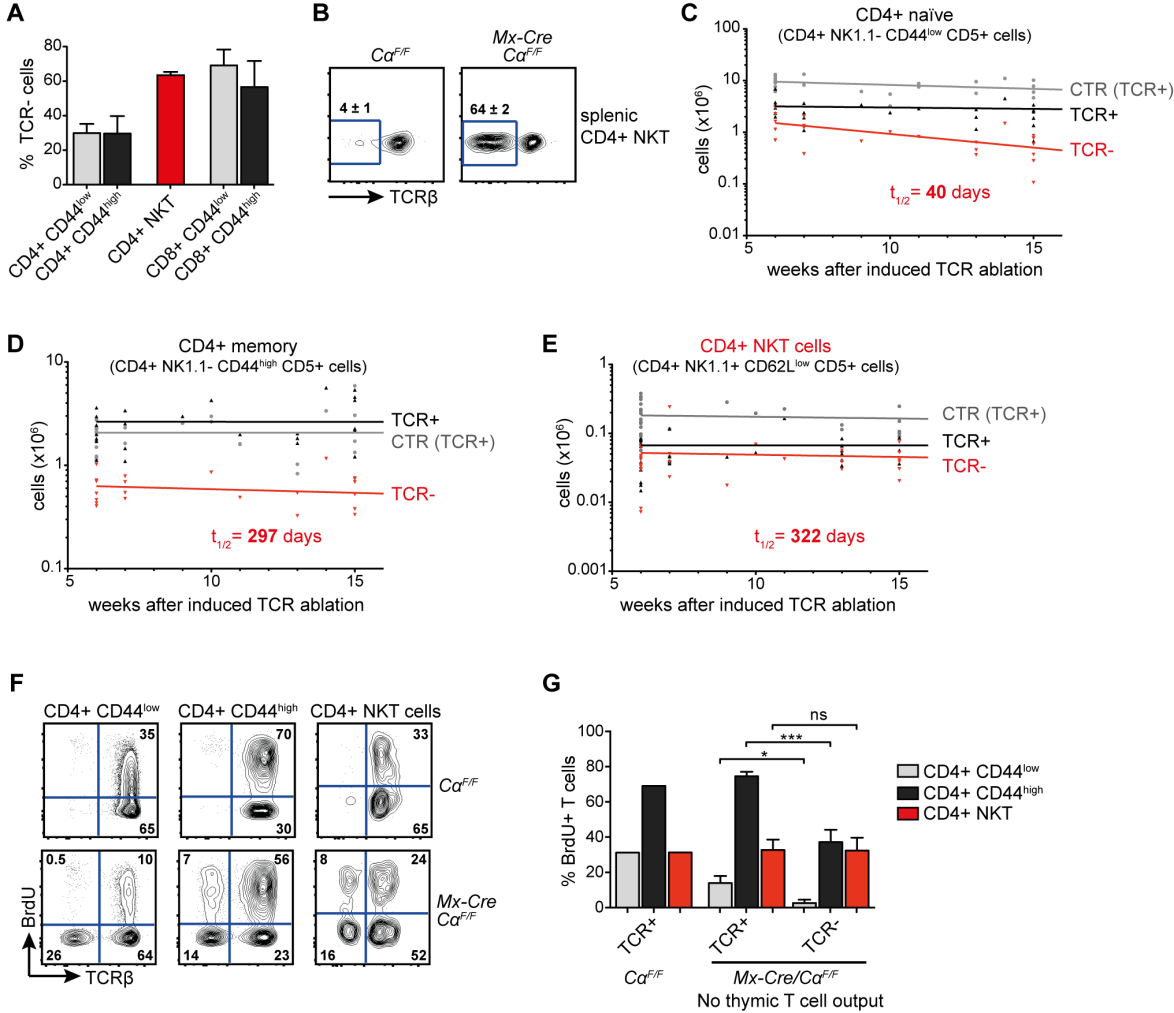
TCR signaling is not required for the steady state homeostasis of mature NKT cells. (A) Percentages of TCRβ− cells of the depicted T cell subsets 2 wk after poly(I:C) injection into *Mx-Cre Cα^F/F^* mice. Bars show means and SD (error bars) of 3–5 mice. (B) Surface TCRβ expression of splenic CD4+ NKT cells (NK1.1+ CD5+ CD62L^low^) 2 wk after poly(I:C) injection. Numbers indicate means ± SD of three independent experiments with altogether five mice per genotype. (C, D) Total cell counts of splenic naïve conventional CD4+ T cells (CD5+ CD44^low^ NK1.1−; C) or of memory/effector-like CD4+ T cells (CD5+ CD44^high^ NK1.1−; D) from 26 control *Cα^F/F^* (CTR, TCR+) animals as well as from 24 *Mx-Cre Cα^F/F^* animals, all after poly(I:C) injection (TCR+, TCR−). (E) Splenic CD4+ NKT cell numbers from in total 32 control *Cα^F/F^* animals (CTR, TCR+) as well as TCRβ+ and TCRβ− CD4+ NKT cell numbers from in total 27 *Mx-Cre Cα^F/F^* animals, at the indicated time after poly(I:C) injection. (C–E) Half-lives were calculated with GraphPad Prism software using nonlinear regression, one-phase decay analysis. (F) BrdU was administered for 4 wk via the drinking water, starting 2 wk after poly(I:C) injection. Directly afterwards, animals were sacrificed and BrdU incorporation was measured by flow cytometry. Representative blots of 2 *Cα^F/F^* and 4 *Mx-Cre Cα^F/F^* mice are shown. (G) Bar chart showing proportion of cells that incorporated BrdU of the indicated T cell subtypes. Bars show means calculated from 2 *Cα^F/F^* and means and SD (error bars) 4 *Mx-Cre Cα^F/F^* mice. *** *p*<0.001; * *p*<0.05; ns, not significant; one-way ANOVA.

Two weeks after induced Cre-mediated recombination, around 30% of CD4 and 65% of CD8 T cells had lost functional TCR expression in these mice (Figure 7A and [40]). To unambiguously identify TCR-deficient NKT cells, we developed a robust staining strategy based on CD4, NK1.1, CD5, and CD62L expression (Figure S3A). This limited us to CD4+ NKT cells, but our staining identified over 50% of the total NKT cell populations in thymus and spleen (Figure S3B). Around 65% of the thus identified NKT cells had lost TCR surface expression 2 wk after Cre induction (Figure 7A,B).

Due to complete Cre-mediated recombination in lymphoid progenitors, T cell development is blocked at the double positive stage in *Mx-Cre Cα^F/F^* mice after induction of Cre [40]. This allowed us to study the T cell decay in the absence of cellular efflux from the thymus. In agreement with previous studies [40],[46], we found that loss of the TCR leads to decay of naïve CD4+ CD44^low^ and memory/effector-like CD4+ CD44^high^ T cells with a half-life of 40 d and 297 d, respectively (Figure 7C,D). Interestingly, we observed essentially no decay of receptor-less NKT cells, with a calculated half-life of 322 d (Figure 7E), and could find significant numbers of TCR-deficient NKT cells even 45 wk after TCR deletion (unpublished data). To evaluate the role of TCR signals during in situ homeostatic proliferation, we administered BrdU for 4 wk via the drinking water, starting 2 wk after induced TCR ablation. Naïve CD4+ CD44^low^ as well as CD4+ CD44^high^ memory/effector-like T cells showed significantly decreased BrdU incorporation in TCR-deficient compared to TCR-expressing cells (Figure 7F,G). In contrast, TCR ablation did not affect NKT cell proliferation (Figure 7F,G). Interestingly, the BrdU incorporation was identical in TCR-deficient CD4+ CD44^high^ T and NKT cells, indicating that in the absence of TCR signals the cytokine-driven expansion of CD4+ CD44^high^ memory/effector-like T and NKT cells is similar (Figure 7F,G). Our results therefore indicate that long-term in situ NKT cell homeostasis is completely independent of TCR-induced signals.

### The TCR Is Dispensable for the Identity and Cytokine-Secretion Ability of Mature NKT Cells

**Figure 8:**
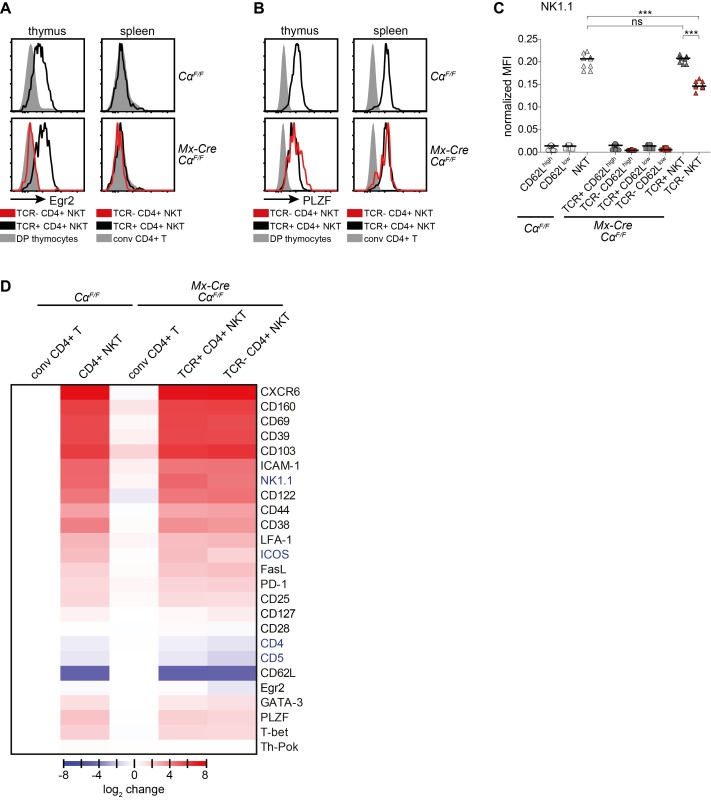
The maintenance of NKT lineage identity does not depend on TCR-signals. *Cα^F/F^* and *Mx-Cre Cα^F/F^* mice were injected with poly(I:C) and analyzed 6 wk later. (A, B) Intracellular expression of Egr2 (A) and PLZF (B) in T cells from the depicted mice. Plots are representative for at least three independent experiments. (C) Flow cytometric analysis of NK1.1 expression on splenic naïve (CD62L^high^ CD5+), memory/effector-like (CD62L^low^ CD5+) CD4+ T cells, and CD4+ NKT cells (NK1.1+ CD5+ CD62L^low^), with or without TCR expression. Median fluorescence intensity, normalized to NK1.1 expression of NK cells (NK1.1+ TCRβ− CD5−). Bars indicate medians. *** *p*<0.001; ** *p*<0.01; * *p*<0.05; ns, not significant; one-way ANOVA. (D) Flow cytometric expression analysis of extra- and intracellular markers of splenic T cells. Median fluorescence intensities of at least four mice per analyzed protein were normalized to the expression on/in conventional CD4+ T cells (tetramer− TCRβ+) to account for interexperimental variations. Expression of NK1.1, CD122, FasL, and T-bet were normalized to NK cells (NK1.1+ TCRβ− CD5−) and then set to 1 for naïve T cells. Data are shown as heatmap, calculated by Perseus software. Blue letters, significantly reduced on splenic TCR− CD4+ NKT cells in comparison to TCR+ CD4+ NKT cells from *Cα^F/F^* and *Mx-Cre Cα^F/F^* mice; analyzed by one-way ANOVA.

In absence of de novo T cell generation, we found elevated Egr2 expression in mature thymic, but not splenic, NKT cells compared to DP thymocytes and CD4+ T cells, respectively (Figure 8A). This indicates that NKT cells receive stronger TCR signals in the thymus, which is supported by the decreased Egr2 expression of mature thymic TCR-deficient NKT cells (Figure 8A). Surprisingly, in mature NKT cells in thymus and spleen, expression of the TCR-signal-induced key transcription factor PLZF is completely unaffected by TCR ablation (Figure 8B).

In order to more generally evaluate to what extent NKT cell TCR-expression is required for the maintenance of characteristic lineage-specific gene expression (resembling recently activated T cells), we extensively analyzed the cell-surface phenotype of NKT cells 6 wk after TCR ablation. Of all the analyzed markers, the only significant changes that we observed on splenic NKT cells upon TCR ablation were downregulation of NK1.1, CD4, CD5, and ICOS (Figures 8C,D and S3C–E). NK1.1 expression was also reduced in thymic TCR-deficient NKT cells, in addition to CXCR6 expression (unpublished data). CD5 and ICOS expression were also reduced in TCR-deficient splenic naïve as well as CD62L^low^ CD4+ T cells (Figure S3C,D). CD4 was upregulated on TCR-deficient CD4+ naïve, but downregulated on NKT and CD4+ CD44^high^ T cells (Figure S3E). Strikingly, all other cell surface markers characteristic for the NKT cell lineage, among them the transcription factors PLZF, GATA-3, T-bet, and Th-POK, as well as many cell surface markers whose expression is also induced upon TCR engagement, remained largely unaffected by loss of the NKT cell TCR (Figure 8D).

**Figure 9:**
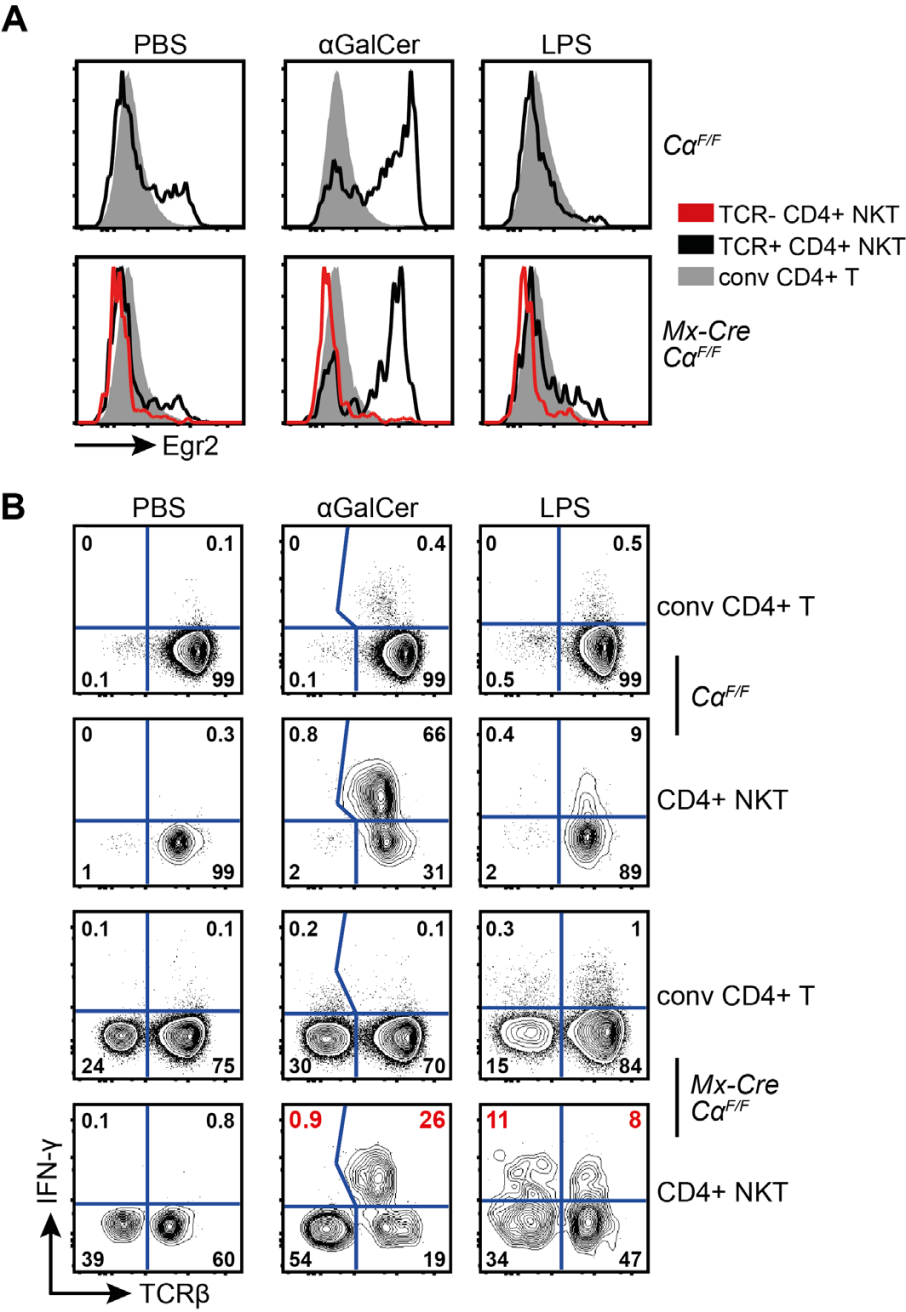
TCR-signals are not required for the innate activation of NKT cells. (A) Intracellular Egr2 expression of the depicted splenic T cells, 90 min after PBS or αGalCer injection, or 6 h after LPS injection. Plots are representative for at least three independent experiments. (B) Intracellular IFN-γ expression of the depicted cells stained directly ex vivo without addition of brefeldin or monensin. Splenic cells were isolated 90 min after PBS or αGalCer injection, or 6 h after LPS injection. Plots are representative for at least three independent experiments.

Treatment of mice with LPS, a cell wall component of gram-negative bacteria, leads to release of IFN-γ by NKT cells via stimulation with IL-12 and IL-18 produced by innate immune cells. This does not require acute TCR engagement [21]. However, it has been proposed that the ability of NKT cells to rapidly release IFN-γ in this context critically requires continuous weak TCR activation in the steady state [25]. We therefore analyzed IFN-γ release of TCR+ and TCR- NKT cells after in vivo injection of LPS, α-GalCer, and PBS (Figure 9A,B). As expected, Egr2 expression could only be detected in NKT cells that were activated through their TCR (Figure 9A). Accordingly, 90 min after α-GalCer injection, the majority of TCR+ NKT cells, but virtually none of the TCR- NKT cells or the CD4+ conventional T cells, produced IFN-γ protein (Figure 9B). Interestingly, NKT cell activation through LPS injection in vivo was able to induce similar IFN-γ production by TCR- NKT cells in comparison to their TCR+ counterparts (Figure 9B). Our results thus clearly demonstrate that homeostasis and key features defining the nature of NKT cells, namely the unique activated cell-surface phenotype and the innate capacity for instant production of IFN-γ, do not require continuous auto-antigen recognition in the mouse.

## Discussion

The elucidation of NKT cell function and their intriguing semi-invariant TCR benefited enormously from Vα14i-TCR transgenic mouse models [11],[32],[47],[48]. Over the last years, it became increasingly clear that premature expression of transgenic TCRα chains, including Vα14i [11],[32], leads to various unwanted side-effects such as impaired β-selection and the generation of large numbers of DN T cells both in the periphery and in the thymus [26],[27]. This drawback affects even TCR alleles generated through nuclear transfer of mature NKT cells [33]. For that reason, Baldwin et al. developed a system in which a transgenic *CAGGS*-promoter-driven TCRα-chain is expressed upon CD4-Cre-mediated excision of a *loxP*-flanked STOP cassette, mimicking the physiologic expression time point [26]. Likewise, Griewank and colleagues expressed the Vα14i-TCR under direct control of *CD4* promoter and enhancer sequences [11]. These are clear improvements, but carry the inbuilt caveats of the respective heterologous expression construct. For example, it has been shown that a large proportion of activated mature T cells loses expression from such transgenic *CD4* promoter enhancer constructs [49].

Here, we present a novel approach, in which the expression of the transgenic Vα14i-TCRα-chain, and in the future any other TCRα-chain of interest, can be initiated via CD4-Cre at the DP stage in the thymus, and is under endogenous control of the *Tcrα* locus throughout the lifespan of the cell. In these mice, large numbers of bona fide CD4+ and DN NKT cells were generated. The reduced proportions of fully mature stage 3 NKT cells (NK1.1+, CD69^high^, T-bet+), as well as the reduced numbers of NK cells, are most likely a consequence of limiting amounts of common differentiation and maintenance factors, such as IL-15 [14],[37],[50]. In addition, attenuated TCR-signaling due to increased competition for self-antigen/CD1d-complexes might delay the full maturation of NKT cells in the transgenic animals. TCR signals have been proposed to play a role in the initiation of CD69 expression on NKT cells, as well as in the induction of IL-2Rβ, the β-chain of the IL-2 and IL-15 receptors [13].

Moreover, we observed the generation of tetramer+ CD8+ T cells. CD8+ NKT cells are found in the human, but not in wild-type mice. CD8 expression on Vα14i NKT cells does not interfere with negative selection, avidity for antigen presented by CD1d, or NKT cell function [28]. Instead, it was proposed that the absence of CD8+ NKT cells in the mouse is due to the constitutive expression of the transcription factor Th-Pok in all CD4+ as well as DN NKT cells [28]. Th-Pok has been shown to be crucial for the maturation and function of NKT cells, and directly represses CD8 expression [28]. This scenario fits well with the fact that the CD8+ tetramer+ T cells in the *CD4-Cre Vα14i^StopF^/wt* (as well as in the Vα11p-*Vα14itg* animals) did not express Th-Pok. These cells also lack many other characteristic features of NKT cells, including PLZF expression. Therefore, we refer to them as tetramer+ CD8+ T cells.

Given the faithful recapitulation of endogenous TCRα-chain expression timing and strength in our knock-in mice, combined with the extremely high homologous recombination efficiency, we believe that our strategy should prove useful for the generation of further novel TCR-transgenic mouse models. By replacing RAG-mediated Vα14 to Jα18 recombination with Cre-mediated activation of Vα14i expression in *CD4-Cre Vα14i^StopF^/wt* mice, we can directly couple conditional gain or loss of gene function with Vα14i-TCR expression in NKT cells. NKT cell-specific gene targeting in mice with physiological NKT cell numbers could be achieved through the generation of mixed bone marrow chimeras with *Jα18^−/−^* bone marrow, which cannot give rise to Vα14i-NKT cells.

Our studies were designed to elucidate whether or to what extent the expression of the autoreactive semi-invariant TCR would activate a peripheral mature naïve conventional T cell, convert it into an NKT cell, or induce gene expression typical of NKT cells. We took advantage of the conditional nature of the Vα14i-TCR knock-in transgene for a TCR switch experiment on conventional peripheral T cells. Naïve CD4+ T cells inherit a high plasticity [51]. Depending on TCR signaling strength and cytokine environment, they can differentiate in various subsets in periphery. This differentiation includes the induction of specific transcription factors, namely T-bet (Th1), GATA-3 (Th2), ROR-γt (Th17), and FoxP3 (peripherally derived regulatory T cells). For NKT cells, it is believed that strong TCR signaling, together with homotypic interactions involving the SLAM family (SLAMf) receptors 1 and 6, ultimately leads to PLZF induction during thymic development [11],[13]. DP thymocytes, presenting auto-antigen via CD1d and also expressing SLAMf members, are crucial for thymic NKT cell selection [11]. These SLAMf receptors are expressed on peripheral lymphocytes in comparable levels to double positive thymocytes (www.immgen.org). Therefore, lymphocytes, especially marginal zone B cells, which express CD1d to a similar level as DP thymocytes, should be able to present antigen and SLAMf-mediated co-stimulation, to naïve conventional T cells with a newly expressed Vα14i-TCR on their surface. The elevated levels of the TCR-induced transcription factor Egr2 in switched tetramer+ T cells suggest that they receive an (auto-)antigenic signal. This finding is in principle in agreement with our finding that tetramer+ TCR-switched T cells are enriched in cells that express Vβ2- and Vβ8.1-/8.2-containing Vα14i-TCRs. These TCRs were shown to have the highest avidity for NKT cell antigens [3]. Furthermore, Vβ7-containing Vα14i-TCRs were shown to be favored when endogenous ligand concentration are suboptimal in CD1d^+/−^ mice [42]. In fact, in CD4+ tetramer+ TCR-switched T cells the relative enrichment for Vβ7-expressing cells was slightly higher than for Vβ2- and Vβ8.1-/8.2-expressing cells (unpublished data). However, the interpretation that this advantage is due to antigenic selection is at odds with the fact that Vβ7-expressing cells are not enriched in tetramer+ TCR-switched CD8+ T cells. We currently have no satisfactory explanation for this discrepancy. Both CD4+ and CD8+ Vα14i-TCR-expressing conventional T cells show features of activation and exhaustion/anergy, but do not develop into NKT cells, judged by absent PLZF and NK cell marker expression. This indicates that either mature T cells have lost the ability to enter the NKT cell lineage, the peripheral Vα14i-TCR signal is not strong enough, or as yet unidentified components of the thymic microenvironment are required to induce an NKT cell fate. Indeed, the high Egr2 expression of mature NKT cells that matured in the periphery and migrated back to the thymus (Figure 8A) suggests that stronger self-antigens are presented at this location. Interestingly, unlike TCR-switched tetramer+ T cells, Egr2 expression in mature splenic NKT cells was similar to that of conventional mature CD4+ T cells. Our data therefore suggest that in the periphery, the Vα14i-TCR can recognize self-lipids, but maturing NKT cells undergo a developmental program that prevents an auto-reactive inflammatory response. At this point, we cannot exclude the possibility that the observed cellular activation was antigen-independent. The fact that the internal control cells, the co-transferred tetramer− T cells, show no or significantly less signs of activation strongly argues for an involvement of antigen recognition or tonic signaling by the Vα14i-TCR. It also remains possible that the transient immune activation caused by the poly(I:C) administration contributes to the observed phenotypes. In all likelihood, this contribution is small, as we never observed any significant immune activation, not to mention loss of CD1d-expressing antigen-presenting B cells and dendritic cells, in *Mx-Cre Cα^F^/wt* control mice that received poly(I:C). Despite these caveats, our results clearly show that under our experimental conditions, Vα14i-TCR expression on conventional naïve T cells leads to their activation and general immune deregulation.

These findings seemed to support notions that NKT cell maintenance [52], their activated surface phenotype, and especially their rapid cytokine expression abilities might depend on constant antigen recognition [25]. However, by ablating the TCR on mature NKT cells in situ, we unequivocally demonstrated that long-term mouse NKT cell homeostasis and gene expression are nearly completely independent of TCR signals. In this regard, they are similar to memory T and B cells, which can maintain their numbers, identity, and functional capabilities in the absence of antigen [53],[54]. Our results are hard to reconcile with a recent report suggesting that NKT cell maintenance requires lipid presentation by B cells [52]. While there might be some differences between mouse and man, a more likely scenario is that the observations of Bosma et al. reflect rather acute local activation than true homeostatic requirements. Most of the known functions of NKT cells critically depend on their ability to rapidly secrete large amounts of many different immune-modulatory cytokines shortly after their activation. Still, it is not fully understood how NKT cell activation is triggered in different disease settings, and especially to what extent signaling in response to TCR-mediated recognition of antigens versus activation by proinflammatory cytokines contributes to this. Various studies reported that CD1d-dependent signals were required for full NKT activation in vitro [19],[20],[55], although most of them contained the caveat of potentially incomplete blockade of CD1d function by blocking antibodies. Our experiments, in line with a recent report [21], show that even in the complete absence of TCR signaling for 4 wk, NKT cells can be robustly activated in vivo to produce IFN-γ upon LPS injection in similar amounts as their TCR+ counterparts. Thus, we demonstrate that in mouse NKT cells continuous steady-state TCR-signaling is not required to maintain the *Ifng* locus in a transcriptionally active state, as recently proposed for human NKT cells [25]. Therefore, our results clearly demonstrate that cellular identity and critical functional abilities of mature NKT cells, such as steady-state proliferation and innate cytokine secretion ability, although initially instructed by strong TCR signals, do not require further antigen recognition through their TCR.

Collectively, our data strongly support the view that Vα14i-TCR expression on developing NKT cells triggers a program that makes them unresponsive to peripheral self-antigens, which can continuously be recognized by their auto-reactive TCR. NKT cells are extremely potent immune-modulatory cells that upon activation can instantly secrete a large array of cytokines. Although they are selected by high affinity to auto-antigens, similar to regulatory T cells, they are not mainly suppressive cells. Therefore, it seems plausible that NKT cells are rendered “blind” to peripheral auto-antigens, rather than depend on continuous stimulation by self-lipids to maintain their cellular identity and innate functions. By keeping their activated state independent of self-antigen recognition, NKT cells can stay poised to secrete immune-activating cytokines while minimizing the risk of causing damage to self during normal physiology. On the other hand, the presence of the auto-reactive Vα14i-TCR serves to detect pathogenic states when a stronger signal is generated by the enhanced presentation of potentially more potent self-antigens or foreign lipids.

## Materials and Methods

### Genetically Modified Mice

To generate *Vα14i^StopF^* mice, B6 ES cells (Artemis) were transfected, cultured, and selected as previously described for Bruce 4 ES cells [56]. *Mx-Cre* [39], *Cα^F^* [40], *CD4-Cre* [57], *Nestin-Cre* [31], Vα11p-*Vα14i-tg* [32], and *Vα14i^StopF^* mice were kept on a C57BL/6 genetic background. As we did not observe any differences between *CD4-Cre* and *Vα14i^StopF^/wt* mice in NKT cell biology, they were sometimes grouped together as controls. Mice were housed in the specific pathogen-free animal facility of the MPIB. All animal procedures were approved by the Regierung of Oberbayern.

### Cre Induction in Mx-Cre Animals

At the age of 6–8 wk (or 2 wk after cell transfer for the TCR switch experiment), animals were given a single i.p. injection (400 µg) of poly(I:C) (Amersham). All mice were analyzed 6–8 wk after injection, unless otherwise indicated.

### Flow Cytometry and Heat Map Generation

Single-cell suspensions were prepared and stained with monoclonal antibodies: B220 (clone RA3-6B2), BTLA (8F4), CD11c (N418), CD122 (TM-b1), CD127 (A7R34), CD160 (eBioCNX46-3), CD25 (PC61.5), CD28 (37.51), CD38 (90), CD39 (24DMS1), CD4 (RM4-5), CD44 (IM7), CD45RB (C363.16A), CD5 (53-7.3), CD62L (MEL-14), CD69 (H1.2-F3), CD8α (53-6.7), CD8β (H35-17.2), CD95 (15A7), DX5 (DX5), Egr2 (erongr2), GATA-3 (TWAJ), Gr1 (RB6-8C5), ICOS (7E.17G9), IL-4 (11B11), IL-13 (eBio13A), IL-17A (eBio17B7), IFN-γ (XMG1.2), LAG-3 (eBioC9B7W), LFA-1 (M17/4), Ly49A/D (eBio12A8), Ly49C/I (14B11), Ly49G2 (eBio4D11), Mac1 (M1/70), NKG2A (16A11), NKG2D (CX5), NK1.1 (PK136), PD-1 (J43), ROR-γt (AFKJS-9), T-bet (eBio4B10), TCRβ (H57-597), Ter119 (TER-119), Th-POK (2POK), and TNF (MP6-XT22) (all from eBioscience). SiglecF (E50-2440) was from BD. TCRβ chains were stained with the mouse Vβ TCR screening panel (BD). PLZF antibody and the CXCL16-Fc fusion were generous gifts from Derek Sant'Angelo and Mehrdad Matloubian, respectively. mCD1d-tetramers were provided by the NIH tetramer core facility. For intracellular transcription factor stainings, cells were fixed and permeabilized with the FoxP3 staining kit (eBioscience). For intracellular cytokine stainings, mice were injected i.v. in the tail vein with 40 µg of LPS (Sigma) or 2 µg αGalCer (Funakoshi) in a total volume of 200 µl PBS. Afterwards, cells were treated according to manufacturer's instructions with the Cytofix/Cytoperm kit (BD). For multiplex measurement of cytokines in the serum, we used the mouse Th1/Th2 10plex Cytomix kit according to manufacturer's instructions (eBioscience). Samples were acquired on a FACSCanto2 (BD) machine, and analyzed with FlowJo software (Treestar). The heat map was generated using perseus (part of the MaxQuant software [58]).

### BrdU Incorporation

Mice were fed with 0.5 mg/ml BrdU (Sigma) in the drinking water for 4 consecutive weeks. Directly afterwards, BrdU incorporation was analyzed with a BrdU Flow Kit (BD).

### ELISA

Serum TNF levels were determined by ELISA as recommended by the manufacturer (BD).

### Quantitative RT-PCR

RNA was isolated (QIAGEN RNeasy Micro Kit) and reverse transcribed (Promega) for quantitative real-time polymerase chain reaction (PCR) using probes and primers from the Universal Probe Library (Roche Diagnostics) according to the manufacturer's instructions.

### Statistics

Statistical analysis of the results was performed by one-way ANOVA followed by Tukey's test, or by student *t* test, in Prism software (GraphPad). The *p* values are presented in figure legends where a statistically significant difference was found.

## Supporting Information

Figure S1 Southern blot screening strategy for the *Vα14i^StopF^* knock-in allele and NKT cell characterization in *Vα14i–* transgenic mice. (A) DNA of targeted neomycin-resistant embryonic stem cells was digested with BamHI. The Southern Blot probe contains the untranslated exon 4 of Cα and recognizes a 12.5 kb fragment for the knock-in in comparison to 8.9 kb for the wild-type allele. Representative Southern blot for 325 clones, six of eight showing homologous integration of the knock-in allele. (B) Absolute cell numbers in thymus and spleen of 7–13 mice of the indicated genotypes of CD8+ tetramer+ T cells. Bars indicate medians. *** *p*<0.001; ns, not significant, one-way ANOVA. (C) CD8α/CD8β expression of splenic CD8α+ NKT cells from *CD4-Cre Vα14i^StopF^/wt* animals. Numbers indicate mean percentages ± SD of three mice. (D) Serum cytokine levels, measured by FlowCytomix, of three CTR mice and each five *CD4-Cre Vα14i^StopF^/wt* and Vα11p-*Vα14itg* mice. (E, F) CD69 and intracellular T-bet expression of NK1.1+/NK1.1− NKT cells from CTR and *CD4-Cre Vα14i^StopF^/wt* mice. Numbers in representative histogram indicate percentage of CD69^high^ or T-bet+ cells among the indicated NKT cells calculated from eight animals per genotype (CD69) or three animals per genotype (T-bet). Histograms are representative of three or more independent experiments with each at least seven mice in total. (G) CD69 expression of CD4+ conventional T cells (filled grey) and NKT cells (black) from Vα11p-*Vα14itg* mice. Number in representative histogram indicates percentage of CD69^high^ cells among the NKT cells, calculated from seven animals. Throughout the figure, NKT cells were gated as tetramer+ TCRβ+, conventional (conv) T cells as tetramer− TCRβ+; CTR, *CD4-Cre* or *Vα14i^StopF^/wt*.
(TIF)

Figure S2 NKT-cell-depletion before cell transfer and additional analysis of the animals in the TCR-switch experiment. (A) Splenocytes of the indicated genotypes were stained before and after depletion of NKT cells by MACS. Numbers indicate percentages of tetramer+ and tetramer− T cells (TCRβ+). Plots are representative for over 15 independent experiments. (B) Staining with “unloaded” mCD1d-tetramer in comparison to PBS57-loaded mCD1d-tetramer of splenocytes from the same animal. Plots are representative for three independent experiments with five mice in total. (C) Expression of intracellular IFN-γ or TNF ex vivo 90 min after αGalCer injection. Data are representative of two independent experiments with two animals each. (D) Representative histograms of flow cytometric analysis of T cells in animals 8 wk after switch induction: CD4+ tetramer–, CD4+ tetramer+, CD8+ tetramer–, and CD8+ tetramer+ T cells (TCRβ+). Surface expression of the depicted markers in comparison to NK cells (gated as marker+, TCRβ−). Representative plots for at least three independent experiments with at least one mouse each.
(TIF)

Figure S3 Gating strategy for the TCR-ablation experiments. (A) Gating strategy to identify TCR− NKT cells. (B) Yield of the applied gating strategy. (C–E) Extracellular expression of the depicted proteins on CD4+ naïve (CD62L^high^ CD5+), CD4+ memory/effector-like (CD62L^low^ CD5+) T cells, and CD4+ NKT cells (NK1.1+ CD5+ CD62L^low^). MFIs were normalized to the expression of CD4+ naïve T cells.
(TIF)

Table S1 Comparison of different Vα14i-transgenic mice.
(DOCX)

## Abbreviations

α-GalCer: α-Galactosylceramide
DN: double-negative
Egr2: early growth response protein 2
GATA-3: GATA binding protein 3
ICOS: inducible T-cell co-stimulator
NKT: natural killer T
PLZF: promyelocytic leukemia zinc finger
ROR-yt: Retinoic acid-related Orphan Receptor Gamma
SLAMf: SLAM family
T-bet: T-box expressed in T cells
tg: transgenic
Th-POK: T-helper-inducing POZ/Krüppel-like factor
Va14i: Vα14-Jα18
