# Supplementary figures and images for: NKT Cell-TCR Expression Activates Conventional T Cells in Vivo, but Is Largely Dispensable for Mature NKT Cell Biology
Source: PLoS Biol. 2013 Jun 18;11(6):e1001589. doi: 10.1371/journal.pbio.1001589 (PMC3708704; doi:10.1371/journal.pbio.1001589)

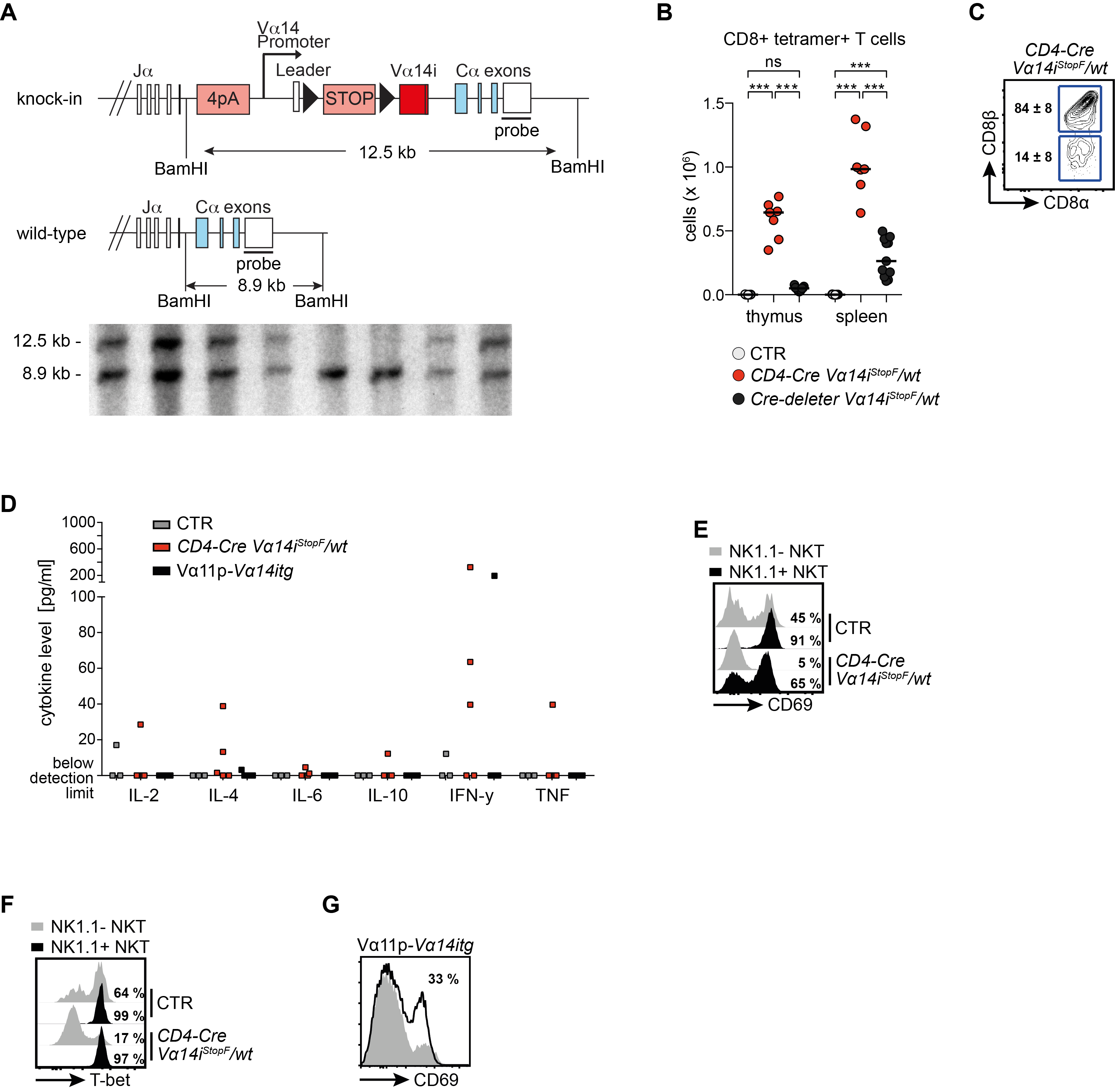

Supplement: Figure S1 — Southern blot screening strategy for the Vα14iStopF knock-in allele and NKT cell characterization in Vα14i– transgenic mice. (A) DNA of targeted neomycin-resistant embryonic stem cells was digested with BamHI. The Southern Blot probe contains the untranslated exon 4 of Cα and recognizes a 12.5 kb fragment for the knock-in in comparison to 8.9 kb for the wild-type allele. Representative Southern blot for 325 clones, six of eight showing homologous integration of the knock-in allele. (B) Absolute cell numbers in thymus and spleen of 7–13 mice of the indicated genotypes of CD8+ tetramer+ T cells. Bars indicate medians. *** p<0.001; ns, not significant, one-way ANOVA. (C) CD8α/CD8β expression of splenic CD8α+ NKT cells from CD4-Cre Vα14iStopF/wt animals. Numbers indicate mean percentages ± SD of three mice. (D) Serum cytokine levels, measured by FlowCytomix, of three CTR mice and each five CD4-Cre Vα14iStopF/wt and Vα11p-Vα14itg mice. (E, F) CD69 and intracellular T-bet expression of NK1.1+/NK1.1− NKT cells from CTR and CD4-Cre Vα14iStopF/wt mice. Numbers in representative histogram indicate percentage of CD69high or T-bet+ cells among the indicated NKT cells calculated from eight animals per genotype (CD69) or three animals per genotype (T-bet). Histograms are representative of three or more independent experiments with each at least seven mice in total. (G) CD69 expression of CD4+ conventional T cells (filled grey) and NKT cells (black) from Vα11p-Vα14itg mice. Number in representative histogram indicates percentage of CD69high cells among the NKT cells, calculated from seven animals. Throughout the figure, NKT cells were gated as tetramer+ TCRβ+, conventional (conv) T cells as tetramer− TCRβ+; CTR, CD4-Cre or Vα14iStopF/wt. (TIF) [file pbio.1001589.s001.tif]

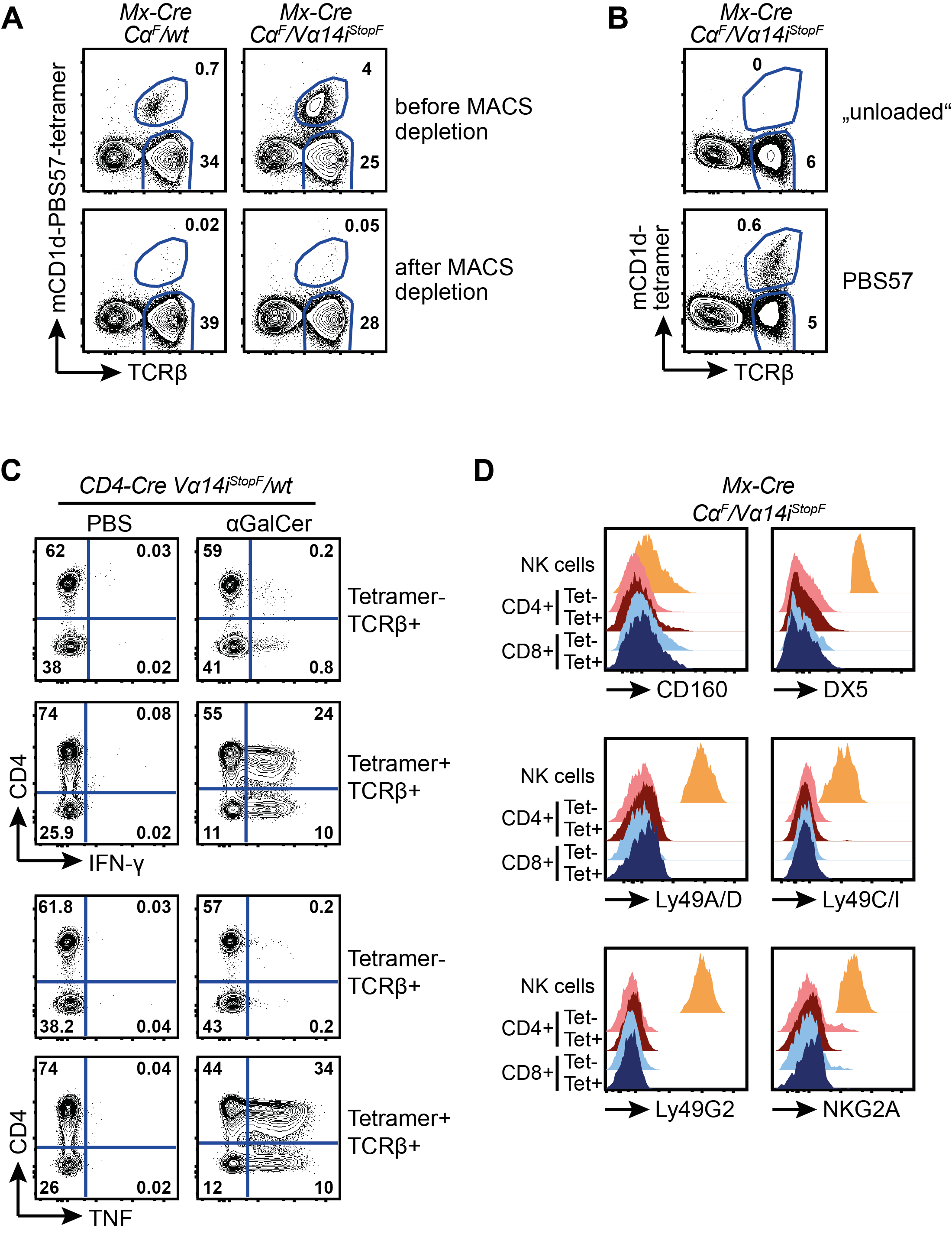

Supplement: Figure S2 — NKT-cell-depletion before cell transfer and additional analysis of the animals in the TCR-switch experiment. (A) Splenocytes of the indicated genotypes were stained before and after depletion of NKT cells by MACS. Numbers indicate percentages of tetramer+ and tetramer− T cells (TCRβ+). Plots are representative for over 15 independent experiments. (B) Staining with “unloaded” mCD1d-tetramer in comparison to PBS57-loaded mCD1d-tetramer of splenocytes from the same animal. Plots are representative for three independent experiments with five mice in total. (C) Expression of intracellular IFN-γ or TNF ex vivo 90 min after αGalCer injection. Data are representative of two independent experiments with two animals each. (D) Representative histograms of flow cytometric analysis of T cells in animals 8 wk after switch induction: CD4+ tetramer–, CD4+ tetramer+, CD8+ tetramer–, and CD8+ tetramer+ T cells (TCRβ+). Surface expression of the depicted markers in comparison to NK cells (gated as marker+, TCRβ−). Representative plots for at least three independent experiments with at least one mouse each. (TIF) [file pbio.1001589.s002.tif]

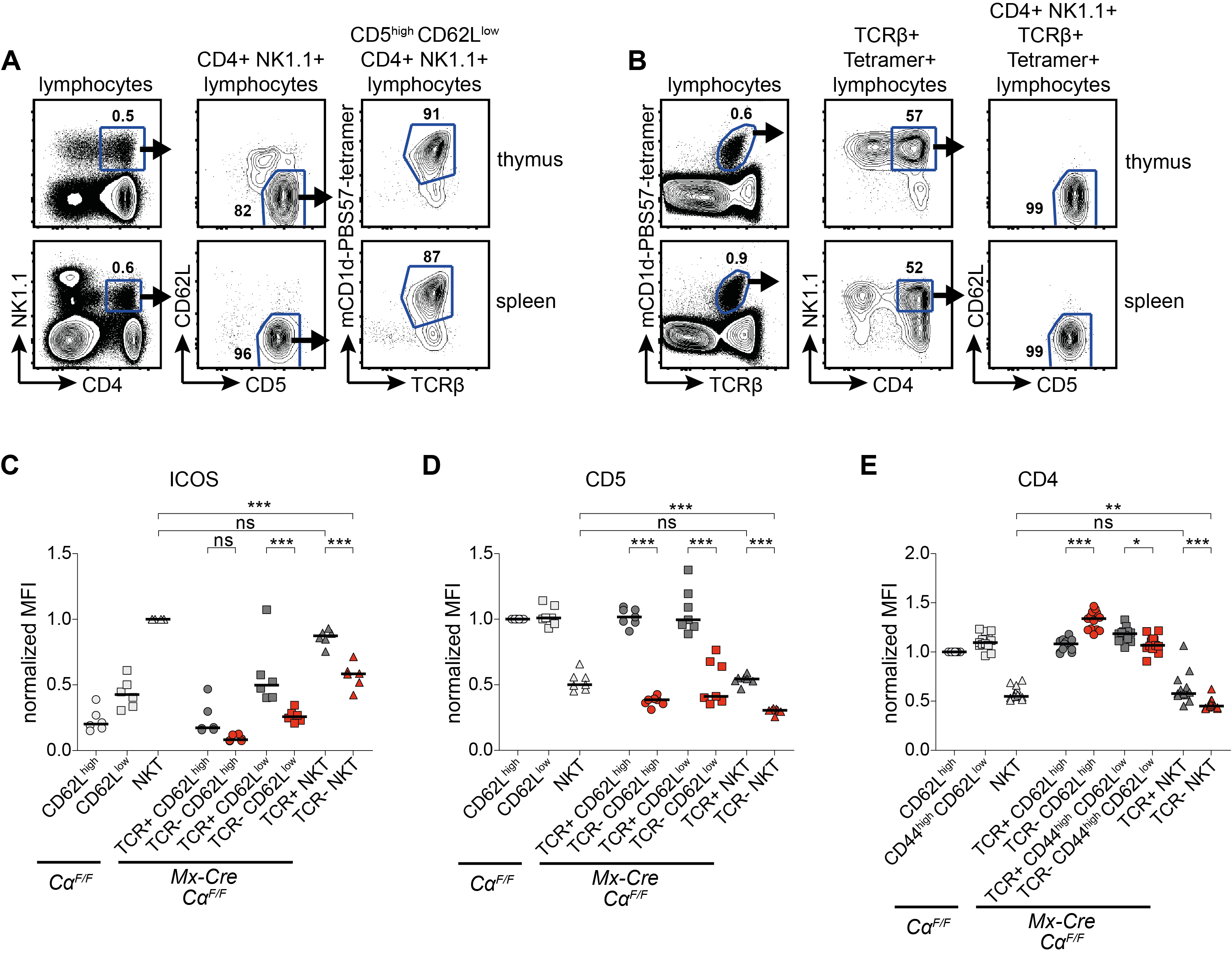

Supplement: Figure S3 — Gating strategy for the TCR-ablation experiments. (A) Gating strategy to identify TCR− NKT cells. (B) Yield of the applied gating strategy. (C–E) Extracellular expression of the depicted proteins on CD4+ naïve (CD62Lhigh CD5+), CD4+ memory/effector-like (CD62Llow CD5+) T cells, and CD4+ NKT cells (NK1.1+ CD5+ CD62Llow). MFIs were normalized to the expression of CD4+ naïve T cells. (TIF) [file pbio.1001589.s003.tif]
