# Supplementary material for: NKT Cell-TCR Expression Activates Conventional T Cells in Vivo, but Is Largely Dispensable for Mature NKT Cell Biology
Source: PLoS Biol. 2013 Jun 18;11(6):e1001589. doi: 10.1371/journal.pbio.1001589 (PMC3708704; doi:10.1371/journal.pbio.1001589)
Supplement: Table S1 — Comparison of different Vα14i-transgenic mice. (DOCX) [file pbio.1001589.s004.docx]

**Table S1: Comparison of different Vα14i-transgenic mice.**

|  | control  (n = 13) | *CD4-Cre Vα14iStop^F^/wt*  (n = 7) | *Cre-deleter Vα14iStop^F^/wt*  (n = 7) | Vα11p-*Vα14itg*  (n = 11) | CD4p-  *Vα14itg*  (Yue et al.) | rec-  *Vα14itg*  (Thapa et al.) | Nuc. transfer-  *Vα14itg*  (Wakao et al.) |
| --- | --- | --- | --- | --- | --- | --- | --- |
| **thymus** |  |  |  |  |  |  |  |
| total cells (x 10^6^) | 132 ± 39 | 54 ± 13 | 15 ± 3 | 15 ± 4 | - | - | 16 ± 10 |
| % NKT cells | 0.4 | 15.2 | 10.4 | 14.8 | 9.0 | 1.1 | 16 |
| total NKT cells (x 10^6^) | 0.3 | **7.9** | 1.7 | 2.1 | 4.0 | - | 2.5* |
| NKT: % CD4+/DN/CD8+ | 68/32/- | 67/24/8 | 44/53/3 | 36/61/4 | - | - | - |
| CD+/DN/CD8+ NKT cells (x 10^6^) | 0.2/0.1/- | 4.6/1.7/0.6 | 0.7/0.9/0.1 | 0.7/1.2/0.1 | - | - | - |
| NKT: % of CD69^high^ | 84 | 61 | - | 54 | - | - | 28 |
| CD69^high^ NKT cells (x 10^6^) | 0.3 | 4.8 | - | 1.1 | - | - | 0.7* |
| NKT: % of NK1.1+ | 87 | 15 | 27 | 24 | 10** | 66 | 23 |
| NK1.1+ NKT cells (x 10^6^) | 0.3 | 1.2 | 0.5 | 0.5 | 0.4*/** | - | 0.6* |
| DN tetramer- T cells (x 10^6^) | 0.1 | 0.1 | 0.5 | 1.0 | - | - | - |
| **spleen** |  |  |  |  |  |  |  |
| total cells (x 10^6^) | 74 ± 19 | 70 ± 14 | 57 ± 19 | 47 ± 7 | - | - | 60 ± 12 |
| % NKT cells | 0.6 | 19.1 | 13.5 | 8.3 | 4.5 | 3.8 | 12.0 |
| total NKT cells (x 10^6^) | 0.3 | **12.1** | 6.1 | 3.7 | 5.7 | - | 7.2* |
| NKT: % CD4+/DNCD8+ | 80/20/- | 72/19/9 | 46/49/5 | 25/66/9 | - | - | - |
| CD+/DN/CD8+ NKT cells (x 10^6^) | 0.2/0.1/- | 8.7/2.2/1.0 | 2.7/2.9/0.3 | 1.0/2.4/0.3 | - | - | - |
| NKT: % of CD69^high^ | 57 | 17 | - | 33 | - | - | - |
| CD69^high^ NKT cells (x 10^6^) | 0.2 | 2.1 | - | 1.2 | - | - | - |
| NKT: % of NK1.1+ | 72 | 20 | 31 | 44 | - | - | - |
| NK1.1+ NKT cells (x 10^6^) | 0.2 | 2.5 | 1.9 | 1.7 | - | - | - |
| DN tetramer- T cells (x 10^6^) | 0.3 | 0.2 | 1.3 | 0.9 | - | - | - |

Shown are average total cell counts and percentages (+/- SD) in thymus and spleen of the indicated mice. Grey results were not measured by us, but instead taken from the respective publications. *, calculated by multiplying the representative percentage with the average cell number. **, probably higher due to dim NK1.1 staining.
